# Non-invasive evaluation of hemodynamics in pulmonary hypertension by a Septal angle measured by computed tomography pulmonary angiography: Comparison with right-heart catheterization and association with N-terminal pro-B-type natriuretic peptide

**DOI:** 10.3892/etm.2013.1324

**Published:** 2013-09-30

**Authors:** QIANG TANG, MIN LIU, ZHANHONG MA, XIAOJUAN GUO, TUGUANG KUANG, YUANHUA YANG

**Affiliations:** 1 Department of Cardiology, Shougang Hospital, Peking University, Beijing 100144, P.R. China; 2 Department of Radiology, Beijing Chaoyang Hospital, Capital Medical University, Beijing 100020, P.R. China; 3 Respiratory Diseases Research Center, Capital Medical University, Beijing 100020, P.R. China

**Keywords:** pulmonary hypertension, computer tomographic pulmonary angiography, N-terminal pro-B-type natriuretic peptide

## Abstract

The septal angle, an angle between the interventricular septum and the line connecting the sternum midpoint and thoracic vertebral spinous process, as measured by computed tomographic pulmonary angiography (CTPA), has been observed to be increased in patients with pulmonary hypertension (PH), but its meaning remains unclear. The aim of this study was to investigate the potential role of the septal angle in evaluating hemodynamics and its association with N-terminal pro-B-type natriuretic peptide (NT-proBNP) in patients with PH. Patients with PH (n=106), including 76 with chronic thromboembolic pulmonary hypertension (CTEPH) and 30 with pulmonary artery hypertension (PAH), were retrospectively reviewed. The patients underwent CTPA prior to right-heart catheterization. The septal angle was measured on transversal CTPA images. Hemodynamic parameters were evaluated by right-heart catheterization. The level of plasma NT-proBNP was measured by enzyme-linked sandwich immunoassay. The septal angle had a moderate correlation with cardiac output (CO; r=−0.535, P=0.000) and a high correlation with pulmonary vascular resistance (PVR; r=0.642, P=0.000). The mean level of NT-proBNP in PH was 1,716.09±1,498.30 pg/ml, which correlated with the septal angle (r=0.693, P=0.000). In a stepwise forward regression analysis, the Septal angle was entered into the final equation for predicting PVR, leading to the following equation: PVR = 28.256 × Septal angle - 728.72. In CTEPH, the Septal angle strongly correlated with NT-proBNP (r=0.668, P=0.000) and PVR (r=0.676, P=0.000). In PAH, the Septal angle strongly correlated with NT-proBNP (r=0.616, P=0.003) and PVR (r=0.623, P=0.000). The CTPA-derived Septal angle is a superior predictor for evaluating and monitoring the level of NT-proBNP and PVR in patients with PH.

## Introduction

Pulmonary hypertension (PH) is defined as a mean pulmonary arterial pressure (mPAP) >25 mmHg at rest or as pulmonary vascular resistance (PVR) >3 Wood units (1). PH comprises apparently heterogeneous conditions that may have comparable clinical and hemodynamic characteristics, and is characterized by progressive obliteration of pulmonary arterioles, leading to the increase of PVR, right-heart failure and mortality. Over the past years, numerous advances in researching and understanding the pulmonary vascular biology have been revealed. The evaluation of the severity of PH is important in the diagnostic process and therapeutic decision-making.

The clinical history, physical examination and biochemical markers of a patient may suggest PH and right ventricular dysfunction. Currently, right-heart catheterization enables the direct measurement of hemodynamics and remains the reference standard for the evaluation of PH (2,3), and it is yet to be replaced by another approach. However, invasiveness and surgical skill requirements are the major weaknesses of heart catheterization. Echocardiography is able to evaluate pulmonary pressure and right ventricular function in a noninvasive manner. However, occasionally echocardiography underestimates the mild increased pulmonary pressure and is unable to supply information concerning emboli of the distal pulmonary artery. B-type natriuretic peptide (BNP) and its prohormone N-terminal pro-B-type natriuretic peptide (NT-proBNP) are peptides released from the myocardium, and studies suggest an abnormal increase in the NT-proBNP response to ventricular wall stress and dysfunction. In patients with PH, increasing evidence suggests that NT-proBNP is a useful marker for right ventricular dysfunction and predicts the outcome of PH (4–7).

Recently, computed tomographic pulmonary angiography (CTPA) has been used to provide much more important information, including establishing the diagnosis, defining its cause, quantifying its hemodynamic parameters, and aiding therapeutic planning and monitoring in pulmonary vascular disease. CTPA is already established as a first line test in acute pulmonary embolism (APE) (8–11). The retrospective electrocardiography (ECG)-gating technique in CT angiography now enables more comprehensive evaluation of cardiac vessels and function. Previously, studies have reported that certain cardiovascular parameters from CTPA are able to assess the extent of pulmonary artery obstruction and right ventricular function (12–15). An angle in transversal images of CTPA between the connecting line from the midpoint of the sternum to the thoracic vertebral spinous process and interventricular septum, named as Septal angle by us firstly, was reported to be correlated with PVR in patients with chronic thromboembolic pulmonary hypertension (CTEPH) (16,17). For further clarification of the role of the Septal angle in assessment severity of PH and its correlation with right ventricular function, we performed a retrospective study to explore the association between the CTPA-derived Septal angle and hemodynamics, and the level of NT-proBNP in patients with PH.

## Materials and methods

### Patients

Between January 2007 and March 2011, 106 consecutive patients with confirmed PH including 76 CTEPH patients and 30 pulmonary artery hypertension (PAH) patients in Peking University Shougang Hospital and Chaoyang Hospital of Capital Medical University (Beijing, China) were included in this retrospective study. PH was diagnosed according to the European Society of Cardiology (ESC)/European Respiratory Society (ERS) Guidelines (2). The medical record of each patient was reviewed by one doctor. Patients who underwent CTPA, right-heart catheterization and NT-proBNP tests were included. Exclusion criteria included the following: i) patients that were not subject to CTPA or CTPA with inferior image quality; ii) patients without right-heart catheterization; and iii) patients without NT-proBNP testing. The control group was composed of 106 age- and gender-matched control subjects without PH and pulmonary embolism. The study protocol was approved by the institutional ethics review committees of Peking University and Captial Medical University. All patients provided written informed consent.

### CTPA acquisition and image analysis

A 64-row multidetector CT scanner (LightSpeed VCT; GE Healthcare, Milwaukee, WI, USA) using the retrospective ECG-gated mode was used for CTPA scanning. The whole chest was scanned from the lung apex to the diaphragm with a single breath-hold. Scan parameters included the following: current of 300–550 mA modulated by personal body mass index (BMI) dose; tube voltage of 100–140 kV and collimation of 0.625 mm, gantry rotation time of 0.8 sec, table speed of 39.37 mm/sec and reconstruction increment of 1 mm. A mechanical injector was used for intravenous bolus injection of iopromide (370 mg/ml, Ultravist; Bayer Schering Pharma, Berlin, Germany) at a flow rate of 4.5–5.0 ml/sec. The automatic bolus-tracking technique had the region of interest positioned at the level of the main pulmonary artery with a pre-defined threshold of 100 HU, and a fixed delay of 5 sec was employed for data acquisition.

**Figure 1:**
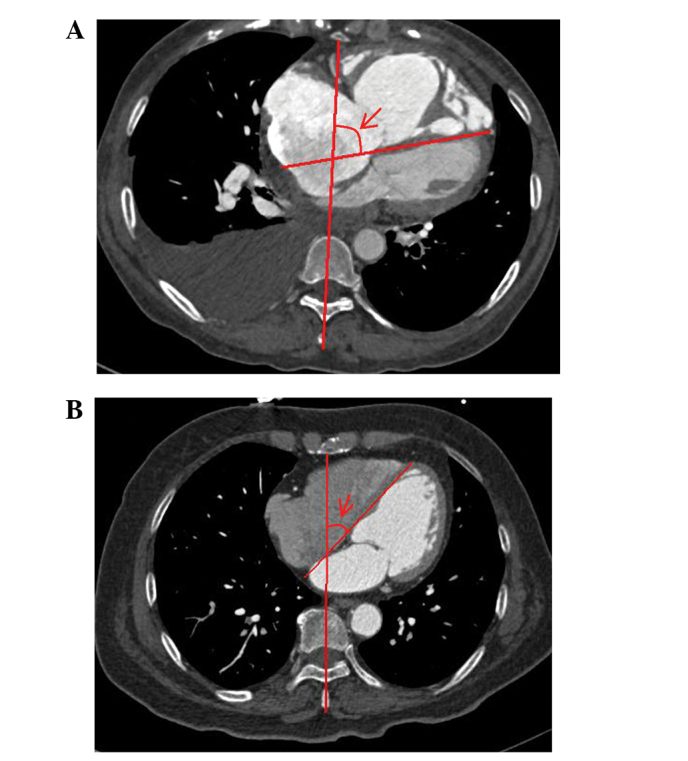
Septal angle measured on transverse images of computed tomographic pulmonary angiography (CTPA) in (A) a patient with CTEPH and (B) a normal patient. CTEPH, chronic thromboembolic pulmonary hypertension.

Images were transferred to the electronic picture archiving and communication systems (GE Centricity 3000 RA1000; GE Healthcare) and reviewed by two radiologists together blinded to clinical information. Fig. 1 shows that the Septal angle was measured in diastole in the transverse CTPA image. According our previously described methods (15–17), other parameters including the diameter of main pulmonary artery (MPAd), diameter of ascending aorta (AAd), transverse diameter of right atrium (RAd), transverse diameter of right ventricle and left ventricle (RVd and LVd), interventricular septal thickness (IVST), and right and left ventricular area (RVa and LVa) were all measured on the transverse images. The ratio of MPAa and AAd (MPAa/AAd), the ratio of RVd and LVd (RVd/LVd) and the ratio of RVa and LVa (RVa/LVa) were measured.

### Right-heart catheterization

Right-heart catheterization was used at 3–5 days after CTPA. By using the Seldinger technique, an 8F Swan-Ganz catheter (Baxter Healthcare, Irvine, CA, USA) was introduced through a right internal jugular vein and positioned under fluoroscopic guidance in a pulmonary artery. After a 10-min rest for stabilization, hemodynamic parameters including right atrial pressure (RAP), pulmonary arterial systolic pressure (sPAP), pulmonary artery diastolic pressure (dPAP), pulmonary capillary wedge pressure (PCWP) and cardiac output (CO) were obtained at end-expiration, then the mPAP and PVR were calculated. The PVR was calculated as described by Tramarin *et al*(18). CO was determined by the thermodilution method (19), with the exception of two cases of congenital heart disease-associated PAH where the Fick method (20) was used to determine CO, as the mean of three consecutive measurements not varying by >10%.

### Plasma level of NT-proBNP

When we performed pulmonary angiography and right-heart catheterization, a blood sample was drawn from the pulmonary artery and immediately transferred into ethylenediaminetetraacetic acid-glass tubes and centrifuged at 3,000 rpm for 15 min at 4°C. The level of NT-proBNP was determined by an enzyme-linked sandwich immunoassay (Elecsys NT-proBNP; Roche Diagnostics, Indianapolis, IN, USA).

### Statistical analysis

All data are expressed as mean ± standard deviation (SD), unless otherwise specified. All analyses were performed with a statistical package (SPSS 13; SPSS, Inc., Chicago, IL, USA). A Mann-Whitney U test was used to compare the Septal angle in the PH and normal control groups. The association of Septal angle with clinical parameters was calculated by univariate analysis. The correlations of Septal angle with NT-proBNP and hemodynamics were analyzed with the Spearman's correlation. Stepwise linear regression analysis was used to evaluate the predictive power of independent CTPA variables for PVR. The receiver-operating characteristic (ROC) method was used to analyze the cut-off value of Septal angle and NT-proBNP for assessing PVR ≥1,000 (dyn.sec/cm^5^). All P-values were for 2-sided tests. P<0.05 was considered to indicate a statistically significant result.

## Results

### Characteristics of the study population

**Figure 2:**
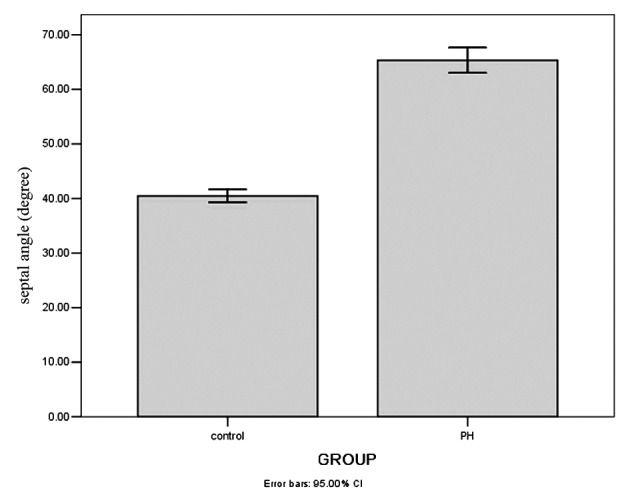
Comparison of Septal angle in pulmonary hypertension (PH) and the control group. CI, confidence interval.

The clinical characteristics of the PH group (age, 14–84 years; median, 51 years) are shown in Table I. The average Septal angle in the PH group was 65.33±11.93° with a range of 40.00–97.30° (Fig. 1A) and all patients had clear signs of PAH as revealed in Table I. In the control group, the average Septal angle was 40.47±6.11° with range of 25.50–54.50° and a median of 40.25° (Fig. 1B). As shown in Fig. 2, the Septal angle in the PH group was significantly higher than that in the control group (Mann-Whitney U test, U=280.5, P=0.000).

**Figure 3:**
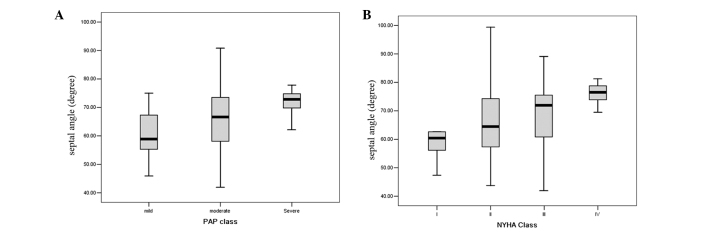
Correlation of Septal angle with (A) class of pulmonary artery pressure (PAP) and (B) New York Heart Association (NYHA) classification.

No correlation was observed between the Septal angle and age (r=0.101, P=0.307), BMI (r=−0.120, P=0.221) or body surface area (r=−0.150, P=0.127). The Septal angle in males and females was 65.86±13.38 and 64.78±10.36°, respectively, which has no significant difference (Mann-Whitney U test; U=1370.0, P=0.959). The Septal angle in CTEPH and PAH groups was 65.76±12.25 and 64.19±11.19°, respectively, and there was no difference in Septal angle between the two groups (Mann-Whitney U test; U=1053.5, P=0.728). The Septal angle was weakly correlated with the class of PAP (r=0.278, P=0.004; Fig. 3A) and New York Heart Association (NYHA) classification (r=0.255, P=0.009; Fig. 3B). No difference in hemodynamic parameters was identified between CTEPH and PAH, as shown in Table II.

### Correlation of the Septal angle with hemodynamics and NT-proBNP in PH

Correlations between the Septal angle and the hemodynamic parameters evaluated by right-heart catheterization (Table III) showed that the Septal angle strongly correlated with PVR (r=0.642, P=0.000), moderately correlated with CO (r=−0.535, P=0.000) and cardiac index (CI; r=−0.534, P=0.000) and weakly correlated with RAP (r=0.255, P=0.009), sPAP (r=0.258, P=0.008), dPAP (r=0.275, P=0.005) and mPAP (r=0.294, P=0.002), but did not correlate with pulse oxygen saturation (SPO_2_; r=0.015, P=0.885) or PCWP (r=−0.025, P=0.803). Correlations between the CTPA variables and PVR (Table IV) suggest that PVR strongly correlated with the Septal angle and moderately correlated with RVa/LVa (r=0.537, P=0.000) and RVd/LVd (r=0.479, P=0.000).

**Figure 4:**
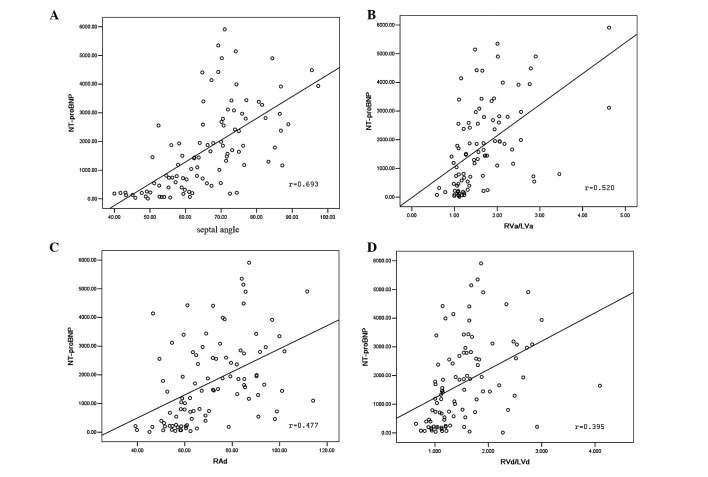
Correlation of computed tomographic pulmonary angiography (CTPA) variables with the level of NT-proBNP. (A) Correlation between Septal angle and NT-proBNP (r=0693, P=0.000); (B) correlation between RVa/LVa and NT-proBNP (r=0.520, P=0.000); (C) correlation between RAd and NT-proBNP (r=0.477, P=0.000); (D) correlation between RVd/LVd and NT-proBNP (r=0.395, P=0.000). NT-proBNP, N-terminal pro-B-type natriuretic peptide; RVa, right ventricular area; LVa, left ventricular area; RAd, transverse diameter of right atrium; RVd, transverse diameter of right ventricle; LVd, transverse diameter of left ventricle.

When all CTPA variables were analyzed in a stepwise forward regression analysis, the Septal angle and RVa/LVa were entered into the final equation for predicting PVR, as shown in Table V, leading to the following two equations: i) PVR = 28.256 × Septal angle - 728.72; and ii) PVR = 23.005 × Septal angle + 207.992 × RVa/LVa - 718.69. The level of NT-proBNP was 1,716.09±1,498.30 pg/ml with a range of 19.79–5,909.00 pg/ml. Correlations between CTPA variables and NT-proBNP (Fig. 4) demonstrated that NT-proBNP strongly correlated with the Septal angle (r=0.693, P=0.000) and moderately correlated with RVa/LVa (r=0.520, P=0.000), RAd (r=0.447, P=0.000) and RVd/LVd (r=0.395, P=0.000).

### Association of Septal angle and hemodynamics, NT-proBNP in CTEPH

In the CTEPH group, Spearman's correlations between the Septal angle and hemodynamic data evaluated by right-heart catheterization (Table III) showed that the Septal angle had a strong correlation with PVR (r=0.676, P=0.000), a moderate correlation with CO (r=−0.586, P=0.000) and CI (r=−0.595, P=0.000), a weak correlation with RAP (r=0.301, P=0.008), dPAP (r=0.251, P=0.029) and mPAP (r=0.256, P=0.026), but had no correlation with SPO_2_ (r=0.022, P=0.853), sPAP (r=0.209, r=0.070) or PCWP (r=−0.053, P=0.655). The level of plasma NT-proBNP in CTEPH was 1,809.52±1,532.16 pg/ml with a range of 19.79–5,909.00 pg/ml, and the Septal angle had a strong correlation with NT-proBNP (r=0.668, P=0.000).

### Correlation of Septal angle and hemodynamics, NT-proBNP in PAH

In the PAH group, Spearman's correlations between Septal angle and hemodynamic data evaluated by right-heart catheterization (Table III) showed that the Septal angle strongly correlated with PVR (r=0.623, P=0.000), moderately correlated with CO (r=−0.553, P=0.000) and CI (r=−0.561, P=0.000), weakly correlated with sPAP (r=0.304, P=0.030), dPAP (r=0.387, P=0.038) and mPAP (r=0.343, P=0.016), but did not correlate with SPO_2_ (r=0.119, P=0.540), RAP (r=0.105, P=0.587) or PCWP (r=−0.221, P=0.249). The level of plasma NT-proBNP in PAH was 1,582.43±1,387.30 pg/ml with a range of 83.10–4,453.00 pg/ml and the Septal angle had a strong correlation with the level of NT-proBNP (r=0.616, P=0.003).

### ROC analysis

**Figure 5:**
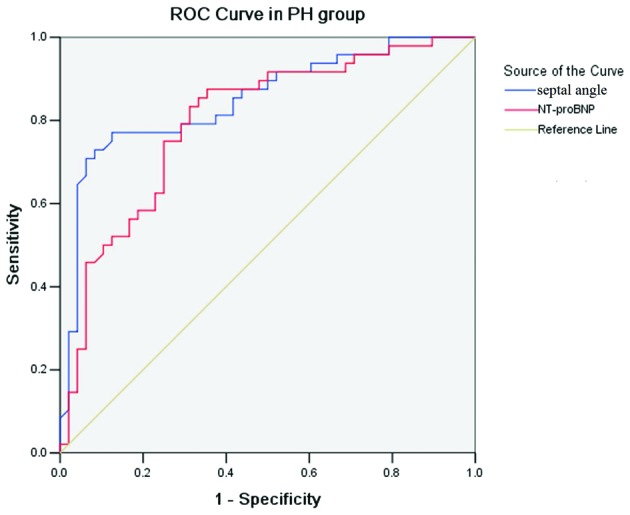
Comparison of receiver-operating characteristic (ROC) curves of Septal angle and NT-proBNP in patients with pulmonary hypertension (PH). The area under the curve (AUC) was 0.850 (95%confidence interval, 0.770–0.929, P=0.000) for Septal angle and 0.808 (95% confidence interval, 0.717–0.905, P=0.000) for NT-proBNP. NT-proBNP, N-terminal pro-B-type natriuretic peptide.

In PH, as shown in ROC analysis (Fig. 5), a Septal angle cut-off point of ≥67.55° had a 77.1% sensitivity and a 87.5% specificity for predicting PVR ≥1,000 (dynes.sec.mm^−5^); its area under the curve (AUC) was 0.850±0.040. An NT-proBNP cut-off point ≥1,443 pg/ml had a 75.0% sensitivity and a 75.0% specificity for predicting PVR ≥1,000 (dynes.sec.mm^−5^); its AUC was 0.808±0.046, comparable or even inferior to the AUC of the Septal angle for predicting PVR ≥1,000 (dynes.sec.mm^−5^).

**Figure 6:**
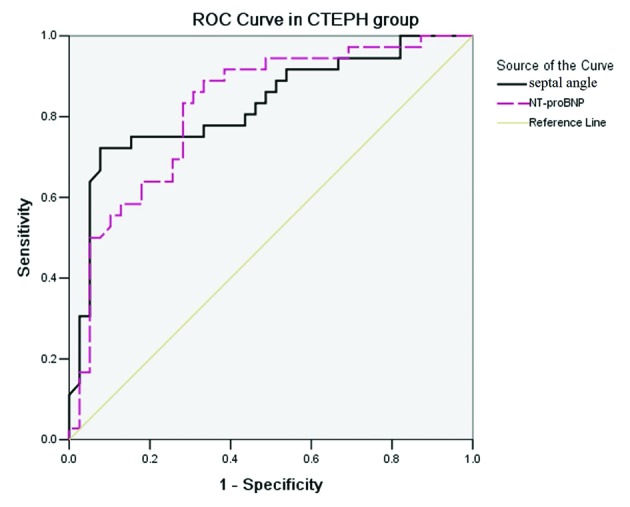
Receiver-operating characteristic (ROC) curves of Septal angle and NT-proBNP predicting pulmonary vascular resistance (PVR) ≥1,000 (dyn.sec/cm^5^) in patients with chronic thromboembolic pulmonary hypertension (CTEPH). The area under the curve (AUC) was 0.827 (95% confidence interval, 0.730–0.924, P=0.000) for Septal angle and 0.822 (95% confidence interval, 0.725–0.918, P=0.00) for NT-pro-BNP. NT-proBNP, N-terminal pro-B-type natriuretic peptide.

In the CTEPH group, ROC analysis (Fig. 6) demonstrated that a Septal angle cut-off point of ≥67.55° had a 75.0% sensitivity and a 84.6% specificity for predicting PVR ≥1,000 (dynes.sec.mm^−5^), and its AUC was 0.827±0.050. An NT-proBNP cut-off point of ≥1,443 pg/ml had a 83.3% sensitivity and a 71.8% specificity for predicting PVR ≥1,000 (dynes.sec.mm^−5^), and its AUC was 0.822±0.049, comparable to the AUC of the Septal angle for predicting PVR ≥1,000 (dynes.sec.mm^−5^).

## Discussion

Our prior studies suggest that CTPA clearly describes the obstructed pulmonary artery (15) and the cardiovascular parameters of CTPA may be used to evaluate the hemodynamics in CTEPH (16,17). In the present study, we analyzed the correlation of Septal angle with the hemodynamics and the level of NT-proBNP in patients with PH and its two subgroups, CTEPH and PAH. We demonstrated that: i) the Septal angle in PH patients is larger than that in the normal control, but weakly correlated with PAP. ii) the Septal angle is strongly correlated with PVR and NT-proBNP in PH and its two subgroups (CTEPH and PAH). iii) In CTEPH, a Septal angle cut-off value of 67.55 has a sensitivity of 77% and a specificity of 81% in predicting PVR ≥1,000 (dyn.sec/cm^5^), comparable to the level of NT-proBNP. iv) In PH, a Septal angle cut-off value of 67.55° is comparable to the NT-proBNP cut-off value of 1,443 pg/ml in predicting PVR ≥1,000 (dyn.sec/cm^5^).

Septal angle is a CTPA-derived parameter that is an angle between the connection line from the midpoint of the sternum to thoracic vertebral spinous process and interventricular septum. This angle represents the balance of left and right ventricular load. Our results revealed that in the control group, the right ventricular (RV) pressure is lower than the left ventricular (LV) pressure; the mean Septal angle is 40.47° (range 25.50–54.50°) and the Septal angle in the PH group is significantly higher than that in the control group, but there is no difference in the CTEPH and PAH groups. This suggests the increased Septal angle is a predictor of PH, and is not affected by the etiology of PH. We observed that Septal angle only weakly increased with the extent of PAP. This suggests PAP does not directly impact on the Septal angle. Although the Septal angle in PH was not affected by age, BMI or body surface area, there was an overlap of Septal angle between PH and the control group, so the normal value of the Septal angle requires validation in a large population. During the progress of PH, the marked increase in PVR limits the rate at which the right ventricle is able to pump blood through the lungs, leading to RV overload, hypertrophy and dilatation (21–24). RV overload causes flattening and leftward displacement of the interventricular septum, and cardiac clockwise rotation, so we suggest that the enlarged septal angle is a sign of the right ventricular overload in patients with PH (23,24).

Resting hemodynamics measured by right-heart catheterization may assess the severity and predict the prognosis of PH (2,3,25). PVR is one of the most important parameters in this, since the increased PVR leads to right ventricular overload and dysfunction. Previous studies demonstrated that parameters such as RVd/LVd correlated with hemodynamic data (16,17). There is no data available so far associating the Septal angle with the resting hemodynamics of patients with PH. We observed a strong correlation of Septal angle and a moderate correlation of RVd/LVd with PVR in PH patients. By a stepwise regression analysis, the Septal angle was entered into a final equation for predicting PVR. This suggests the Septal angle may be a superior indicator for evaluating PVR than RVd/LVd. NT-proBNP is released from myocytes and may be used as an indicator of the severity of PH (26). The very high serum NT-proBNP concentrations observed in the patients with PH who were studied most likely result from an increased RV wall stretch and marked hypertrophy of the RV walls (27–29). Our data indicated that the Septal angle strongly correlated with NT-proBNP and demonstrated that the Septal angle may also be a superior predictor of severity and RV dysfunction in patients with PH.

PVR was critical in the assessment of CTEPH for its importance in the prediction of potential candidates for pulmonary endarterectomy and postoperative outcome (30) and NT-proBNP has been used as a noninvasive marker of the severity of right ventricular dysfunction in CTEPH (31). Although we did not analyze the threshold of the Septal angle in predicting the increased PVR and NT-proBNP in a large population, our results showed that the Septal angle strongly correlated with PVR and NT-proBNP in the CTEPH group. Jamieson *et al*(32) reported that patients with a preoperative PVR >1,000 (dynes.sec.mm^−5^) had a significantly higher mortality rate than those with a preoperative PVR <1,000 (dynes.sec.mm^−5^), so we selected 1,000 (dynes.sec.mm^−5^) as the threshold of PVR. We assessed the value of the Septal angle in predicting PVR ≥1,000 (dynes.sec.mm^−5^). Using ROC curve analysis, Septal angle >67.55° demonstrated superior sensitivity and specificity with a higher AUC to identify PVR ≥1,000 (dynes.sec.mm^−5^) and there was no difference in the AUC between the Septal angle and NT-proBNP level, suggesting that Septal angle and NT-proBNP have similar value in predicting a PVR ≥1,000 (dynes.sec.mm^−5^). These findings may be of practical importance for patients with CTEPH, CTPA not only demonstrates the distribution of clots but also supplies the information of the operability of pulmonary endarterectomy and predicts the survival of patients.

Hemodynamic data and biomarkers aid the diagnosis and assessment of PAH (33). Mukerjee *et al*(34) showed that NT-proBNP levels correlated significantly with PVR in scleroderma patients with PAH. Souza *et al*(35) showed NT-proBNP levels had a high correlation with hemodynamic parameters, particularly PVR in patients with idiopathic pulmonary artery hypertension (IPAH). Our study also showed that the Septal angle was strongly correlated with CO, PVR and NT-proBNP in PAH patients, suggesting that the severity of PAH or right heart strain as measured by hemodynamic data may be estimated using the Septal angle. However, further observation is required due to the relatively small number in this group. Fijalkowska *et al*(6) demonstrated that a NT-proBNP cut-off point at ≥1,400 pg/ml was useful in identifying PH patients with poor long-term prognosis. Notably, using ROC analysis, we observed a serum NT-proBNP level of ≥1,433 pg/ml has a similar value to a Septal angle of ≥67.5° for predicting the PVR ≥1,000 (dynes.sec.mm^−5^) in PH and CTEPH patients, suggesting the predictive value of a Septal angle ≥67.5° may also be used to evaluate the long-term prognosis in PH patients.

Although the present observations indicate that the CTPA-derived Septal angle is a useful parameter for evaluating the severity and RV dysfunction in PH, there are possible limitations. First, the retrospective design of our study allows for less generalization from our results, so although the Septal angle correlated with PVR and NT-proBNP, the correlations require further verification by prospective observations. Secondly, although the Septal angle appears to be useful in evaluating hemodynamics in patients with CTEPH, it is unclear whether operability and surgical success, defined as mortality and/or improvement of PVR, may be predicted with sufficient accuracy since only 14 patients underwent PEA. Our observations require verification from future studies of larger numbers of patients to determine the usefulness of preoperative CTPA in identifying RV dysfunction in high-risk CTEPH patients and the association with postoperative hemodynamic outcome, RV failure and mortality. Thirdly, the Septal angle appears to be useful in evaluating the severity of PH and a Septal angle ≥67.5° also may be used to evaluate the long-term prognosis. However, the lack of prognostic data in a follow-up period may be considered a major limitation of this study. The cut-off value of septal angle is able to indicate the right ventricular function and may assess the prognosis of patients with pulmonary hypertension. However, this would require a larger and homogeneous cohort, considering the numerous therapeutic options available at present.

In summary, our results suggest that the measurement of the Septal angle by CPTA is simple, noninvasive and may be a useful parameter to assess the severity and RV dysfunction in PH and its two subgroups.

## Figures and Tables

**Table I:** Clinical and hemodynamic characteristics of patients with PH.

Characteristics	Values
Baseline parameters (mean ± SD)	
Age (years)	50.86±13.40
Gender (male/female)	53/53
Body mass index (kg/m^2^)	23.44±3.12
Body surface area (m^2^)	1.73±0.18
NT-proBNP (pg/ml)	1716.09±1498.30
Septal angle (degree)	65.32±11.93
Clinical classification of PH (n)	
CTEPH	76
Idiopathic PAH	24
Connective tissue diseases associated	
PAH	2
Heritable PAH	2
PAH associated with congenital heart disease	2
Class of PAP (n)	
Mild (25–39 mmHg)	17
Moderate (40–69 mmHg)	70
Severe (≥70 mmHg)	19
NYHA classification (n)	
I	6
II	44
III	46
IV	10
Hemodynamics (mean ± SD)	
RAP (mmHg)	7.78±6.11
sPAP (mmHg)	86.14±23.70
dPAP (mmHg)	33.11±12.63
mPAP (mmHg)	51.58±15.65
CO (l/min)	3.47±1.36
PCWP (mmHg)	9.13±3.72
PVR (dynes.sec.mm^−5^)	1121.09±582.37

PH, pulmonary hypertension; NT-proBNP, N-terminal pro-B-type natriuretic peptide; CTEPH, chronic thromboembolic pulmonary hypertension; PAH, pulmonary arterial hypotension; PAP, pulmonary artery pressure; NYHA, New York Heart Association; RAP, right atrial pressure; sPAP, pulmonary artery systolic pressure; dPAP, pulmonary artery diastolic pressure; mPAP, mean pulmonary artery pressure; CO, cardiac output; PCWP, pulmonary capillary wedge pressure; PVR, pulmonary vascular resistance.

**Table II:** Comparison of Septal angle and hemodynamics in CTEPH and PAH.

Parameters	CTEPH	PAH	U^a^	P-value
Septal angle (°)	65.76±12.25	64.19±11.19	1053.5	0.728
RAP (mmHg)	8.04±5.90	7.13±6.68	993.5	0.303
sPAP (mmHg)	85.62±20.61	91.00±30.24	1030.0	0.440
dPAP (mmHg)	31.71±9.97	36.67±17.40	921.0	0.124
mPAP (mmHg)	50.00±12.73	55.56±21.08	939.0	0.158
PCWP (mmHg)	8.79±2.97	9.96±5.12	1054.0	0.612
CO (l/min)	3.42±1.22	3.62±1.36	1033.0	0.453
PVR (dynes.sec.mm^−5^)	1113.66±564.96	1139.93±673.60	1106.0	0.812

a Mann-Whitney U test.

CTEPH, chronic thromboembolic pulmonary hypertension; PAH, pulmonary arterial hypertension; RAP, right atrial pressure; sPAP, pulmonary artery systolic pressure; dPAP, pulmonary artery diastolic pressure; mPAP, mean pulmonary artery pressure; PCWP, pulmonary capillary wedge pressure; CO, cardiac output; PVR, pulmonary vascular resistance.

**Table III:** Correlations between Septal angle and hemodynamic parameters in PH and its subgroups.

	Spearman's correlation analysis (r, P-value)		
	
Hemodynamics	PH (n=106)	CTEPH (n=76)	PAH (n=30)
RAP	0.255, 0.009	0.301, 0.008	0.105, 0.587
sPAP	0.258, 0.008	0.209, 0.070	0.304, 0.030
dPAP	0.275, 0.005	0.251, 0.029	0.387, 0.038
mPAP	0.294, 0.002	0.256, 0.026	0.343, 0.016
PCWP	−0.025, 0.803	−0.053, 0.655	−0.221, 0.249
CO	−0.535, 0.000	−0.586, 0.000	−0.553, 0.000
CI	−0.534, 0.000	−0.595, 0.000	−0.561, 0.000
PVR	0.642, 0.000	0.676, 0.000	0.623, 0.000
SPO_2_	0.015, 0.885	0.022, 0.853	0.119, 0.540

PH, pulmonary hypertension; CTEPH, chronic thromboembolic pulmonary hypertension; PAH, pulmonary arterial hypertension; RAP, right atrium pressure; sPAP, pulmonary artery systolic pressure; dPAP, pulmonary artery diastolic pressure; mPAP, mean pulmonary artery pressure; PCWP, pulmonary capillary wedge pressure; CO, cardiac output; CI, cardiac index; PVR, pulmonary vascular resistance; SPO_2_, pulse oxygen saturation.

**Table IV:** Correlations between CTPA variables and PVR in patients with PH.

CTPA parameters	Correlation coefficient^a^	P-value
Septal angle	0.642	0.000
RVa/LVa	0.537	0.000
RVd/LVd	0.479	0.000
RAd	0.321	0.010
RVa	0.248	0.013
RVd	0.240	0.016
MPAd/AAd	0.217	0.027
IVST	−0.139	0.163

a Spearman's correlation analysis.

CTPA, computed tomography pulmonary angiography; PVR, pulmonary vascular resistance; PH, pulmonary hypertension; RVa/LVa, ratio of right ventricular area to left ventricular area; RVd/LVd, ratio of transverse diameters between right ventricle to left ventricle; RAd, transverse diameter of right atrium; RVa, right ventricular area; RVd, transverse diameter of right ventricle; MPAd/AAd, ratio of the diameter of main pulmonary artery to the diameter of the ascending aorta; IVST, interventricular septal thickness.

**Table V:** Stepwise linear regression analysis of the relation between CTPA predictions and PVR.

Model^a^	R	Predictors^b^	F-value	B	t	Significance
Model 1	0.588	Septal angle	52.351	28.256	7.235	0.000
		(constant)		−728.740	−2.811	0.000
Model 2	0.631	Septal angle	32.363	23.005	5.504	0.000
		RVa/LVa		207.992	2.905	0.005
		(constant)		−718.690	−2.784	0.005

a Dependent variable: PVR;

b predictors in the model of ratio of right ventricular area to left ventricular area (RVa/LVa).

CTPA, computed tomography pulmonary angiography; PVR, pulmonary vascular resistance; B, coefficient.
